# Utility of isolated-check visual evoked potential technique in dysthyroid optic neuropathy

**DOI:** 10.1007/s00417-023-05975-9

**Published:** 2023-01-16

**Authors:** Ban Luo, Rong Liu, Shanluxi Wang, Weikun Hu, Yunping Li, Boding Tong, Hong Zhang, Xin Qi

**Affiliations:** 1 Department of Ophthalmology, Tongji Hospital, Tongji Medical College, Huazhong University of Science and Technology, Qiaokou District, No.1095, Jiefang Avenue, Wuhan, 430030 Hubei China; 2 Department of Ophthalmology, the Second Xiangya Hospital, Central South University, No.139, Renmin Middle Road, Changsha, 410011 Hunan China; 3 Department of Ophthalmology, Wenchang People’s Hospital, Wenchang, 571321 China

**Keywords:** Dysthyroid optic neuropathy (DON), Thyroid-associated ophthalmopathy (TAO), Isolated-check visual evoked potential (icVEP), Diagnosis

## Abstract

**Purpose:**

To analyze the utility of isolated-check visual evoked potential (icVEP) for discriminating between eyes with dysthyroid optic neuropathy (DON) and eyes with thyroid-associated ophthalmopathy (TAO) but not DON.

**Methods:**

Forty-three eyes with TAO but not DON (as non-DON), fifty-three eyes with DON, and sixty healthy eyes (as controls) were included. Comprehensive ophthalmic examinations, including best-corrected visual acuity, refraction, color vision test, intraocular pressure measurement, slit-lamp biomicroscopy, ophthalmoscopy, RAPD, exophthalmometry measurements, pVEP test, icVEP test, standard automated perimetry, and clinical activity score classification of TAO, as well as demographic information, were collected and analyzed.

**Results:**

In the DON group, the signal-to-noise ratio (SNR) value of icVEPs decreased significantly compared with that of the non-DON group as well as control (*p* < 0.05). The SNR values under 8%, 16% and 32% depth of modulation (DOM) were significantly negatively correlated with BCVA (*p* < 0.05, *r* =  − 0.9 ~  − 0.6), papilledema (Y/N) (*p* < 0.05, *r* =  − 0.8 ~ 0.4) and DON (Y/N) (*p* < 0.001, *r* =  − 0.7 ~  − 0.5). The 8% DOM of icVEP had the largest area under the receiver operating characteristic curve (AUC) (0.842) for discriminating DON from non-DONs. Meanwhile, decision curve analysis (DCA) showed that patients clinically benefit most from 8% DOM of icVEP. Furthermore, the 8% DOM of icVEP combing with papilledema (Y/N) and BCVA (Model 1) has significantly larger AUC than the 8% DOM of icVEP (*p* = 0.0364), and has better clinical benefit in DCA analysis.

**Conclusions:**

The SNR of 8% DOM from icVEP may represent a significant ancillary diagnostic method for DON detection. Furthermore, icVEP combined with papilledema (Y/N) and BCVA should be considered as a diagnostic model in future clinical practice.

**Supplementary Information:**

The online version contains supplementary material available at 10.1007/s00417-023-05975-9.


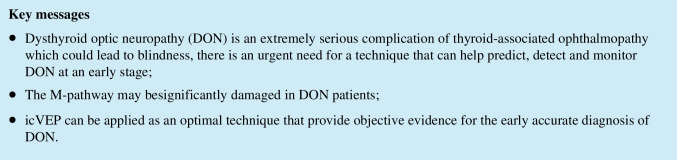


## Introduction

Dysthyroid optic neuropathy (DON) is a relatively uncommon but extremely serious complication of thyroid-associated ophthalmopathy (TAO) [1], it has an estimated incidence of 5% to8.6% [1–5]. The exact pathogenesis of DON remains unclear, but it may be closely related to mechanical compression from retrobulbar soft tissues [5]. As an immune-mediated orbital inflammation, hypertrophied extraocular muscle and fat, as well as neighboring orbital inflammation markedly increased orbital, especially apical tissue pressure, lead directly to optic nerve stretching and restriction of blood supply [2, 5, 6]. Without timely diagnosis and accurate intervention, DON patients have a risk of long-term visual function impairment, which makes prompt and accurate prediction and management essential to reduce morbidity and improve prognosis [1].

DON often presents with decreased visual acuity (VA), impaired color vision, relative afferent pupillary defect (RAPD), papilledema [7], visual field (VF) defect, and abnormal visual evoked potentials (VEP) [6, 8]. However, once these clinical manifestations are discovered, the disease may have progressed to a serious degree of DON and may be irreversible [9].Thus, there is an urgent need for a technique that can help predict, detect and monitor DON at an early stage.

Isolated-check VEP (icVEP) is a novel technique that prone to records steady-state visual evoked potentials and offers an objective measurement of visual functions preferentially reflecting the function of the Magnocellular pathway (M-pathway) [10, 11]. In humans, M cells have relatively large-diameter axons [11] that could be preferentially damaged in optic neuropathy; thus, icVEP has been widely applied in early diagnosis of visual disorders, such as glaucoma [11–14] and traumatic optic neuropathy [15], but never for DON. Given the effectiveness of this technique, we conducted this study to test the hypothesis that [1] the M-pathway is significantly damaged in DON patients and [2] icVEP can be applied as an optimal technique that can provide objective evidence for the early diagnosis of DON.

## Methods

### Study subjects

This was a prospective case–control study, and was approved by the Ethics Committee of the Tongji Hospital of Huazhong University of Science and Technology, and it adhered strictly to the principles of the Declaration of Helsinki. All the subjects were recruited from Tongji Hospital and signed informed consent forms prior to participation.

The diagnoses of TAO and the severity classifications were made using the European Group on Graves’ Orbitopathy criteria [7, 16]. TAO patients meeting the following criteria were included in the study: [1] age > 18 years, [2] clear refractive media allowing sufficient image quality, and [3] no treatment with systemic glucocorticoids for at least 3 months prior to the study. The exclusion criteria for all subjects were as follows: [1] any systemic diseases other than thyroid disorders, [2] any history of ocular surgery or ocular trauma, [3] any ophthalmic diseases other than TAO (e.g., primary glaucoma, diabetic retinopathy, high myopia, and uveitis), and [4] any neurological abnormalities that could account for VF changes. DON was diagnosed based on the presence of two or more of the following clinical findings: relative afferent pupillary defect (RAPD) when unilaterally affected, color visual defect, decreased VA, papilledema, VF defect (mean deviation [MD] in Humphrey perimetry, <  − 1.0 dB), abnormal pattern VEP (pVEP) test (latency delay or/and amplitude reduction).

### Ophthalmic and systemic examination

Each subject enrolled received comprehensive ophthalmic examinations, including best-corrected visual acuity (BCVA), refraction, color vision (pseudoisochromatic plate) test, intraocular pressure (IOP) measurement (noncontact tonometer), slit-lamp biomicroscopy, direct ophthalmoscopy, RAPD (swinging light) test, and exophthalmometry measurements (Hertel exophthalmometer). Subjects with binocular disease were selected with the more severe eye, which was evaluated by BCVA, and if the BCVA of both eyes was the same, the eye with worse VF will be enrolled for the study. The controls were randomly selected with one eye. The clinical activity of TAO was graded according to the Clinical Activity Score (CAS) [3] classification. Automated refraction, VEP test (Huzhou Medconova Medical Technology, Inc.), and standard automated perimetry (SAP, Humphrey Field Analyzer 740i, Carl Zeiss Meditec, Inc. Dublin, CA, USA) were performed. SAP was performed with 24–2 standard procedure using standard test parameters after all the subjects obtained refractive correction with refractometry. The mean deviation (MD) <  − 1.0 dB and pattern standard deviation (PSD) significant at *p* < 0.05 were considered abnormal. pVEP test were using a black-and-white reversing checkboard stimulus (80% contrast, 85 cd/m^2^ luminance) subtending 50’ of arc at the subject’s eye at 50 cm the delayed P100 latent time or/and reducted P100 amplitude were considered abnormal. All subjects underwent more than one VF test to consider learning effects, and only the results of the second reliable VF test [17] were used in the analysis. The reliability criteria of visual field test results are that the fixed loss rate is less than 20%, and the false positive rate and false negative rate are less than 33%. Demographic information was collected for all patients, including age, sex and medical history, such as hyperthyroidism, radioiodine (I131) therapy history and smoking history.

### Isolated-check visual evoked potential (icVEP)

The icVEP was performed using the Neucodia visual electrophysiological diagnostic system (MKWH BMD, Huzhou Medconova Medical Technology, Inc.). Both eyes of the subjects were consecutively tested under natural pupil size. The operation process was followed according to our previous study [18], and the objective monitoring of fixation is achieved by two aspects: the operation time is 2 s, and the infrared monitoring system is used to automatically monitor the fixation. We used swept-parameter stimuli with five increasing levels of the depth of modulation (DOM) of the check luminance: 2, 4, 8, 16, and 32% at duration of 2 s each with the signal-to-noise ratio (SNR) calculated, the first two stimuli (2 and 4%) were included for adaptation purposes. The SNR is a measure of estimated VEP response magnitude relative to the noise (response variability), and it was the final number used to determine the presence of DON.

### Decision curve analysis (DCA)

DCA [19] is a curve drawn by the risk threshold as the abscissa and the net return as the ordinate. The risk threshold is an important parameter when the model is used for decision making, and when this threshold is exceeded, interventions will be taken for the individual. In many prediction studies, researchers generally choose the threshold with the largest sum of sensitivity and specificity as the optimal threshold, but this method is calculated under the assumption that sensitivity and specificity are equally important. However, in actual decision-making, the determination of the risk threshold needs to be considered in all aspects, not only the performance of the prediction model under this threshold, but also the cost and damage of the intervention measures. Therefore, people’s attention to true positives and false positives is inconsistent, that is, the weight coefficients is different. In the forecasting model, this weight factor is the “profit and loss ratio”. The profit and loss ratio is the odds ratio of the risk threshold. Net benefit (NB) is a composite metric that incorporates both true positives and false positives into the calculation. [NB = (NTP- NFP*R)/NT; NTP: the number of true positive people; NFP: the number of false positive people; R: profit and loss ratio; NT: the number of true positive people] A predictive model is more valuable for clinical decision-making when it achieves the maximum NB over a broad range of risk thresholds.

### Statistical analysis

All the analyses were performed using Empower(R) (http://www.empowerstats.com, X and Y solutions, Inc, Boston, MA, USA) and R (http://www.R-project.org). The Kolmogorov–Smirnov test was used to assess the normality of the distribution of continuous data. The differences in non-normally distributed continuous variables between two groups were compared using Mann–Whitney *U*‑tests, and categorical variables were compared using the χ^2^ test or Fisher’s exact test. In addition, generalized estimation was used to adjust the relationship between icVEP and the parameters (e.g., VF, pVEP, et al.), and univariate correlation analysis was performed to assess the associations between various clinical factors and the icVEP parameters. The receiver operating characteristic (ROC) curves were constructed using bootstrap resampling (times = 500) and used to determine the discriminatory capabilities of the tests. The area under the ROC curve (AUC), sensitivity and specificity were evaluated for different parameters. MedCalc (version 16.4.3, Ostend, Belgium; www.medcalc.be) was used to compare the AUCs of each model and indicator in a pair-by-pair fashion. We also used DCA [19] to assess the clinical utility of different diagnostic models for decision-making. The prediction probability is calculated by a regression model using a linear combination of multiple indicators through multivariate logic. Except where otherwise indicated, continuous data are presented as the mean ± standard deviation (SD). *P*-values < 0.05 were considered statistically significant.

## Results

In this study, we included 156 participants in the final analysis, which included 43 TAO but not DON (abbreviated as non-DON) eyes (25 females and 18 males), 53 DON eyes (23 females and 30 males) and 60 healthy (control) eyes (30 females and 30 males). There was a slight age imbalance between DON and non-DON group, and there were no significant differences among groups regarding sex, right/left eye, refraction, degree of exophthalmos, color vision, current thyroid condition, active phase, history of I131 treatments, or history of smoking (Table 1). There were significant differences among the three groups in visual functions, such as BCVA, history of decreased VA, papilledema, RAPD, VF defect, and pVEP.

**Table 1:** Demographic characteristics and visual functions of the study subjects

	Control (*n* = 60)	Non-DON(*n* = 43)	DON (*n* = 53)	*P*-value
Age (year)	51.3 ± 8.3	47.7 ± 8.8	53.2 ± 6.2	0.052/0.410/0.002*
Sex (male/female)	30/30	18/25	30/23	0.414/0.483/0.151†
Eyes(R/L)	30/30	22/21	26/27	0.907/0.920/0.837†
IOP (mmHg)	17.0 ± 2.5	18.8 ± 4.8	19.6 ± 4.4	0.074/**0.002**/0.586*
SE(D)	− 0.3 ± 1.2	− 0.4 ± 1.4	− 0.1 ± 0.9	0.885/0.637/0.402*
BCVA (LogMAR)	0.01 ± 0.03	0.02 ± 0.06	0.21 ± 0.23	> 1.000/** < 0.0001**/** < 0.0001**‡
P100 Amplitude(μV)	9.9 ± 4.5	10.3 ± 6.4	7.6 ± 4.7	0.924/0.056/0.037*
P100 latent time(ms)	101.4 ± 4.8	105.3 ± 6.3	115.4 ± 13.7	0.083/** < 0.0001**/ < 0.0001*
PSD(VF)	2.2 ± 0.9	2.2 ± 1.6	4.7 ± 2.9	0.998/** < 0.0001**/ < 0.0001*
MD(VF)	− 2.1 ± 1.7	− 1.7 ± 1.3	− 6.1 ± 5.7	0.848/** < 0.0001**/ < 0.0001*
Color vision abnormal (Y/N)	0/60	0/43	2/51	> 0.999/0.218/0.500†
RAPD (Y/N)	0/60	0/43	4/49	> 0.999/**0.046**/0.126†
Decreased VA (Y/N)	0/60	2/41	31/22	0.171/** < 0.0001**/** < 0.0001**†
Papilledema (Y/N)	0/60	0/43	13/40	> 0.999/** < 0.0001**/** < 0.0001**†
SNR(8%DOM)	3.3 ± 1.5	1.4 ± 0.6	0.8 ± 0.4	** < 0.0001**/** < 0.0001**/0.009*
SNR(16%DOM)	2.3 ± 1.4	1.7 ± 0.9	0.9 ± 0.7	**0.021**/** < 0.0001**/0.001*
SNR(32%DOM)	2.7 ± 1.4	2.2 ± 1.4	1.3 ± 0.9	0.147/** < 0.0001**/0.0009*
Hyperthyroidism(Y/N)	-	35 /8	45/8	0.646†
I131 therapy(Y/N)	-	11/32	9/44	0.302†
Smoking history(Y/N)	-	15 /28	23/30	0.396†
Active phase(Y/N)	-	18/25	26/27	0.482†
CAS (0/1/2/3/4/5/6/7)	-	5/10/10/6/8/4/0/0	3 /13/11/3/11/6/4/2	0.345†
Duration(m)	-	6.4 ± 4.4	4.6 ± 1.9	0.080§
Exophthalmos(mm)	-	19.0 ± 3.4	19.4 ± 2.6	0.381§

^*^One-way ANOVA Tukey’s multiple comparison test, ‡ Kruskal–Wallis test with Dunn’ test, †Pearson’s Chi-squared test with Yates’ continuity correction, §Mann–Whitney *U*-test

*BCVA*, best-corrected visual acuity; *MAR*, minimum angle of resolution; *VF*, visual field; *PSD*, pattern standard deviation; *MD*, mean deviation; *RAPD*, relative afferent pupillary defect; *SNR*, signal-to-noise ratio; *DOM*, depth of modulation of the check luminance; *pVEP*, pattern visual evoked potentials; *DON*, dysthyroid optic neuropathy; *CAS*, clinical activation score; *IOP*, intraocular pressure; *SE*, spherical equivalent

*P* values: control vs. non-DON / control vs. DON / non-DON vs. DON, values with statistical significance (*P* < 0.05) are in bold

### icVEP examinations

For the three groups, the SNR values at each contrast level were normally distributed; comparisons of the SNRs among groups are provided in Table 1. These data show statistically significant differences among the control, non-DON and DON groups. The SNRs in the DON group decreased significantly compared with those in the non-DON group as well as control group at each contrast level (all *p* values < 0.05).

### Factors associated with SNR (8%, 16%, 32% DOM)

After adjusting for age, sex, refraction, history of smoking, treatment of I131, generalized estimation equations were used to analyze the correlations between the SNR values of icVEPs under the 8%, 16%, and 32% DOM and clinical factors in non-DON and DON groups (Table 2). The SNR values under 8%, 16% and 32% DOM were significantly strongly negatively correlated with BCVA (*p* = 0.038, *r* =  − 0.6; *p* < 0.001, *r* =  − 0.7; *p* < 0.001, *r* =  − 0.9), papilledema (Y/N) (*p* = 0.006, *r* =  − 0.4; *p* = 0.002, *r* =  − 0.6; *p* = 0.010, *r* =  − 0.8) and DON (Y/N) (*p* < 0.001, *r* =  − 0.5; *p* < 0.001, *r* =  − 0.6; *p* < 0.001, *r* =  − 0.7). The SNR values under 8%, 16% and 32% DOM were significantly weakly positively correlated with MD(dB) (*p* = 0.033, *r* = 0; *p* = 0.041, *r* = 0; *p* = 0.004, *r* = 0.1) and significantly weakly negatively correlated with P100 latent time (ms) (*p* = 0.032, *r* =  − 0.0; *p* < 0.001, *r* =  − 0.0; *p* = 0.004, *r* =  − 0.0). The SNR value under 8% DOM was significantly weakly negatively correlated with Degree of exophthalmos (mm) (*p* = 0.011, *r* =  − 0.0));The SNR value under 16% DOM was significantly strongly negatively correlated with Color vision (*p* = 0.017, *r* =  − 0.6); The SNR value under 32% DOM was significantly weakly negatively correlated with PSD(dB) (*p* = 0.021, *r* =  − 0.1). The SNR values were negatively correlated with color vision(8% and 32%DOM), RAPD, PSD (dB) (8% and 16%DOM), duration (m), degree of exophthalmos (mm) (16% and 32%DOM), IOP (mmHg), and CAS, and positively correlated with P100 amplitude (mv), no significant differences were found.

**Table 2:** Factors associated with different contrast of SNRs of icVEP

	SNR(8%DOM)		SNR(16%DOM)		SNR(32%DOM)	
	*r*	*P*-value	*r*	*P*-value	*r*	*P*-value
Duration (m)	− 0.0	0.135	− 0.0	0.257	− 0.1	0.089
Degree of exophthalmos (mm)	− 0.0	**0.011**	− 0.1	0.062	− 0.1	0.061
IOP (mmHg)	− 0.0	0.400	− 0.0	0.273	− 0.0	0.264
CAS	− 0.0	0.263	− 0.1	0.160	− 0.1	0.052
BCVA (logMAR)	− 0.6	**0.038**	− 0.7	** < 0.001**	− 0.9	** < 0.001**
Color vision	− 0.0	0.800	− 0.6	**0.017**	− 0.4	0.305
Papilledema (Y/N)	− 0.4	**0.006**	− 0.6	**0.002**	− 0.8	**0.010**
RAPD (Y/N)	− 0.2	0.200	− 0.1	0.839	0.1	0.887
MD (dB)	0.0	**0.033**	0.0	**0.041**	0.1	**0.004**
PSD (dB)	− 0.0	0.326	− 0.1	0.214	− 0.1	**0.021**
P100 amplitude (mv)	0.0	0.321	0.0	0.129	0.0	0.349
P100 latent time (ms)	− 0.0	**0.032**	− 0.0	** < 0.001**	− 0.0	**0.004**
DON(Y/N)	− 0.5	** < 0.001**	− 0.6	** < 0.001**	− 0.7	** < 0.001**

*IOP*, intraocular pressure; *CAS*, clinical activation score; *BCVA*, best corrected visual acuity; *RAPD*, relative afferent pupillary defect; *PSD*, pattern standard deviation; *MD*, mean deviation; *DON*, dysthyroid optic neuropathy

Values with statistical significance (*P* < 0.05) are in bold

### Diagnostic power of VF, pVEP, and icVEP

**Fig. 1:**
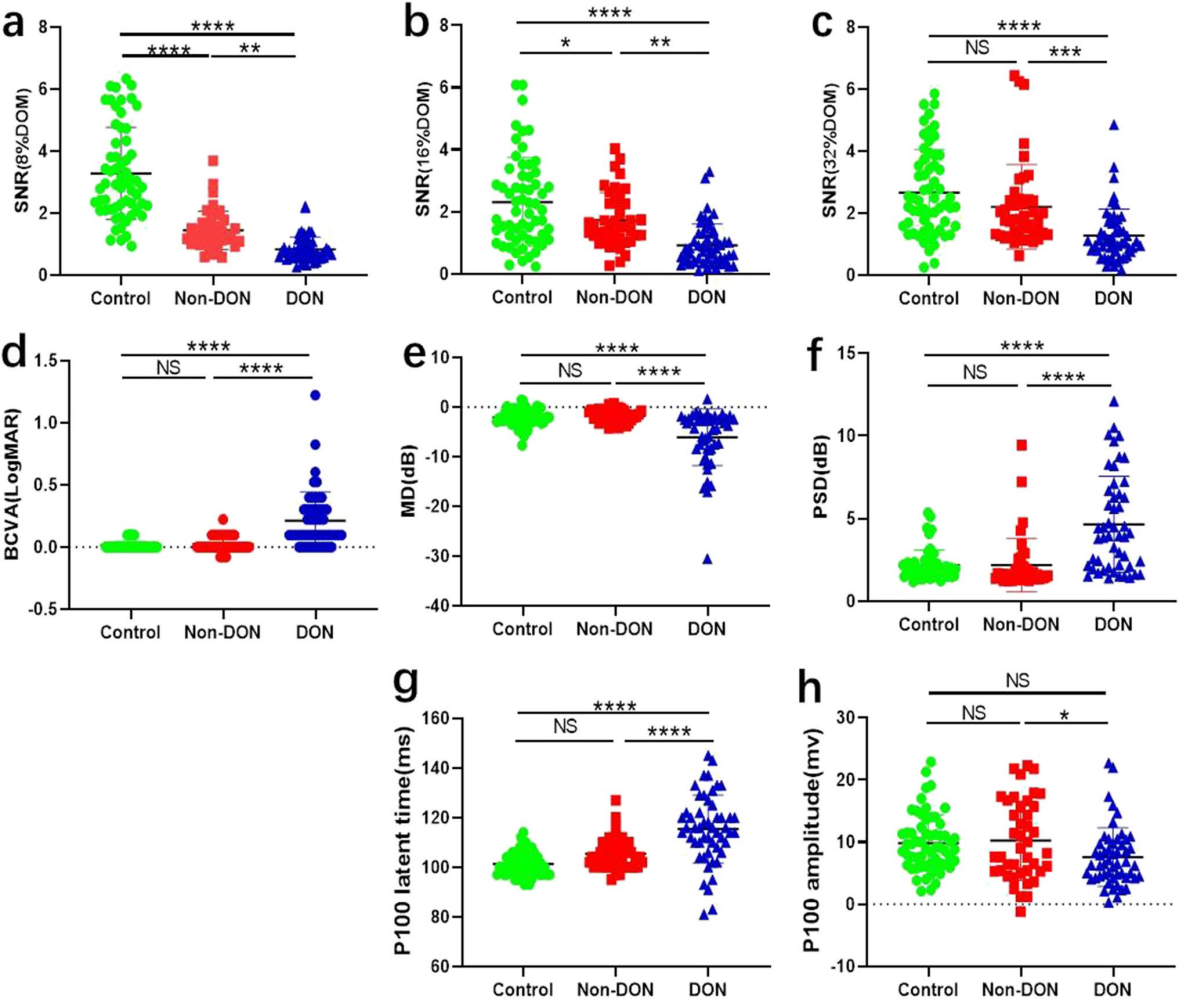
The correlation of the visual functions among three groups. The Scatter plot graphs present the visual function among the control group, DON group and Non-DON group. (**a**–**c**) The value of SNR (8%DOM,16%DOM and 32%DOM) of icVEP in the three groups. (**d**) BCVA in the three groups. (**e**–**f**) The two parameters (MD and PSD) of visual field examination in three groups. (**g**–**h**) The two parameters (P100 Amplitude and P100 latent time) of p-VEP examination in three groups. *p < 0.05; **p < 0.01; ***p < 0.001; ****p < 0.0001

**Fig. 2:**
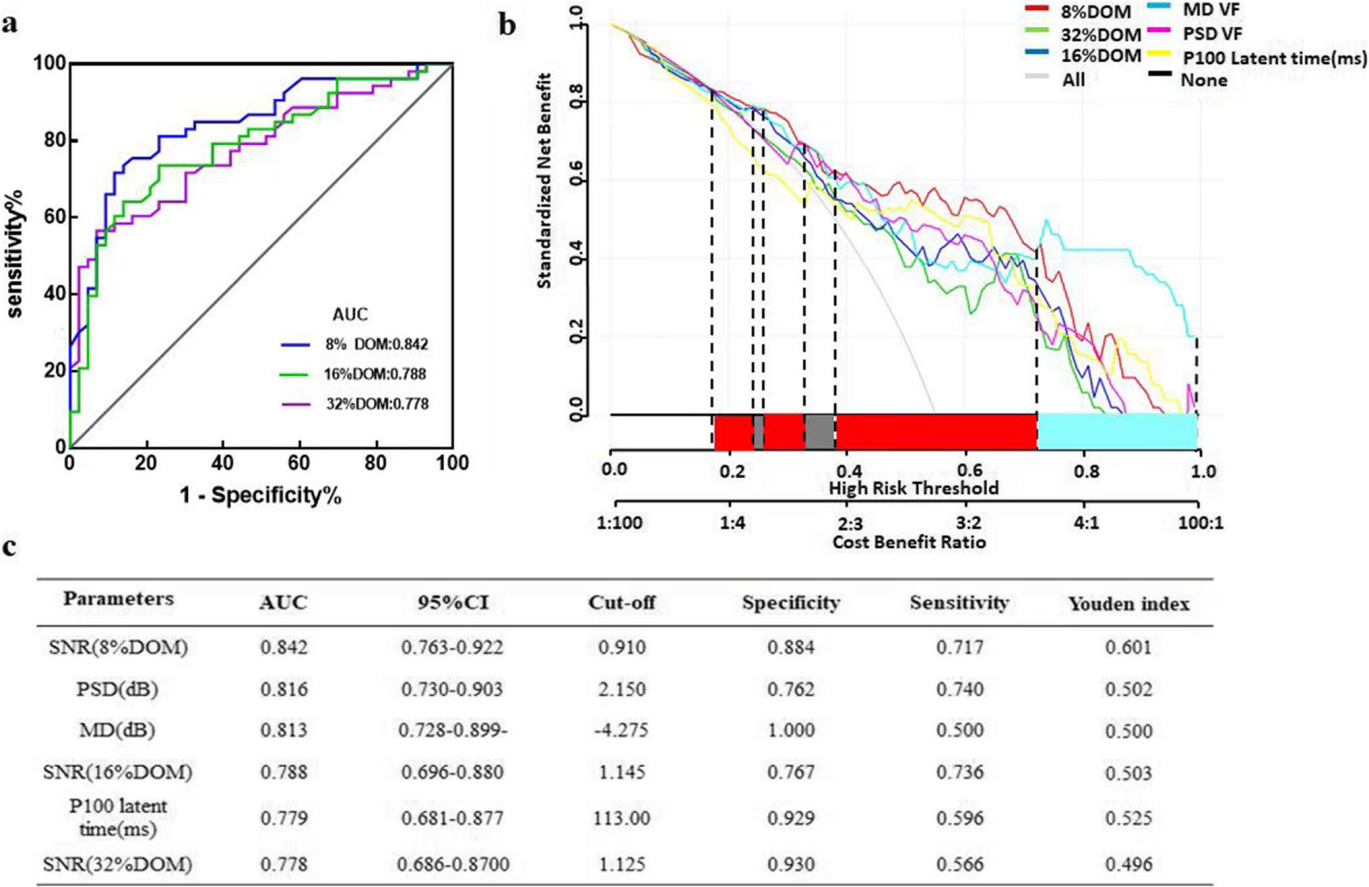
Comparison of icVEP, VF, and pVEP in the diagnosis of DON. (**a**) Receiver operating characteristic curves for the 8%, 16%, and 32% DOM of icVEP. The AUCs for the 8%DOM, 16%DOM and 32%DOM were 0.842 (95% confidence interval [CI], 0.763–0.922); 0.788 (95% confidence interval [CI], 0.696–0.880) and 0.778 (95% CI, 0.686–0.870), respectively. (**b**) Decision curve analysis of six parameters for diagnosing DON. The diagonal light-gray solid line is shown when all patients are considered as having the outcome (which is DON), while the horizontal dark-gray solid line presents when all are considered as not having the outcome; the above two lines represent two extreme cases. On the abscissa line, the red square represents the high risk threshold range with highest NB of the red line (SNR 8%DOM), and the light blue square represents the high risk threshold range of the light blue line (MD VF) with highest NB, while the gray square indicates that multiple DCA curves partially overlap or basically overlap the high risk threshold range. The red curve (8%DOM) has widest range with highest NB. (**c**) Diagnostic power of the SNR (8%DOM, 16%DOM, 32%DOM) of icVEP, PSD(dB), MD(dB), and P100 latent time(ms). The 8% DOM had the largest AUC (0.842) for discriminating DON from non-DON subjects SNR, signal-to-noise ratio; DOM, depth of modulation of the check luminance; PSD, pattern standard deviation; MD, mean deviation

Aiming to find an optimal method for DON detection, we selected the factors that are strongly associated with DON based on Table 1 and analyzed the diagnostic accuracy of VF, pVEP, and icVEP separately. Figure 1 presents that there were no significant differences between control and non-DON on BCVA, MD, PSD, P100 latent time, and P100 amplitude; however, statistical significance was found between the above two groups on SNR values under 8% and 16%. Furthermore, the AUC values of all parameters were greater than 0.7, indicating that all the parameters in Fig. 2a achieved average diagnostic efficacy [20]. Among the measured parameters (Fig. 2c), the 8% DOM had the largest AUC (0.842) and Youden index (0.601) for discriminating DON from non-DON subjects. However, there was no significant difference among the AUC values of all parameters (Supplementary Table 1), indicating that the diagnostic efficacies of icVEP, VF (PSD, MD), and pVEP (P100 latent time) shown no significant difference when diagnosing DON.

In order to testify the clinical utility of these techniques, we applied DCA for further analysis. For the six parameters (SNR values of 8%, 16%, 32% DOM, PSD, MD, P100 latent time), the net benefit (NB) curves are shown in Fig. 2b. The diagonal light-gray solid line is shown when all patients are considered as having the outcome (which is DON), while the horizontal dark-gray solid line presents when all are considered as not having the outcome; the above two lines represent two extreme cases. The abscissa is the threshold rate, and the ordinate indicates the NB after pros and cons. The preferred parameter is the one with the highest NB within a widest threshold range. Thus, according to Fig. 2b, though most curves are reasonably clustered together, the net benefit of 8% DOM SNR at most threshold probabilities (> 50%) (the red threshold range) is greater than other diagnostic parameters indicates its better diagnostic performance.

### Diagnostic power of the prediction models

**Fig. 3:**
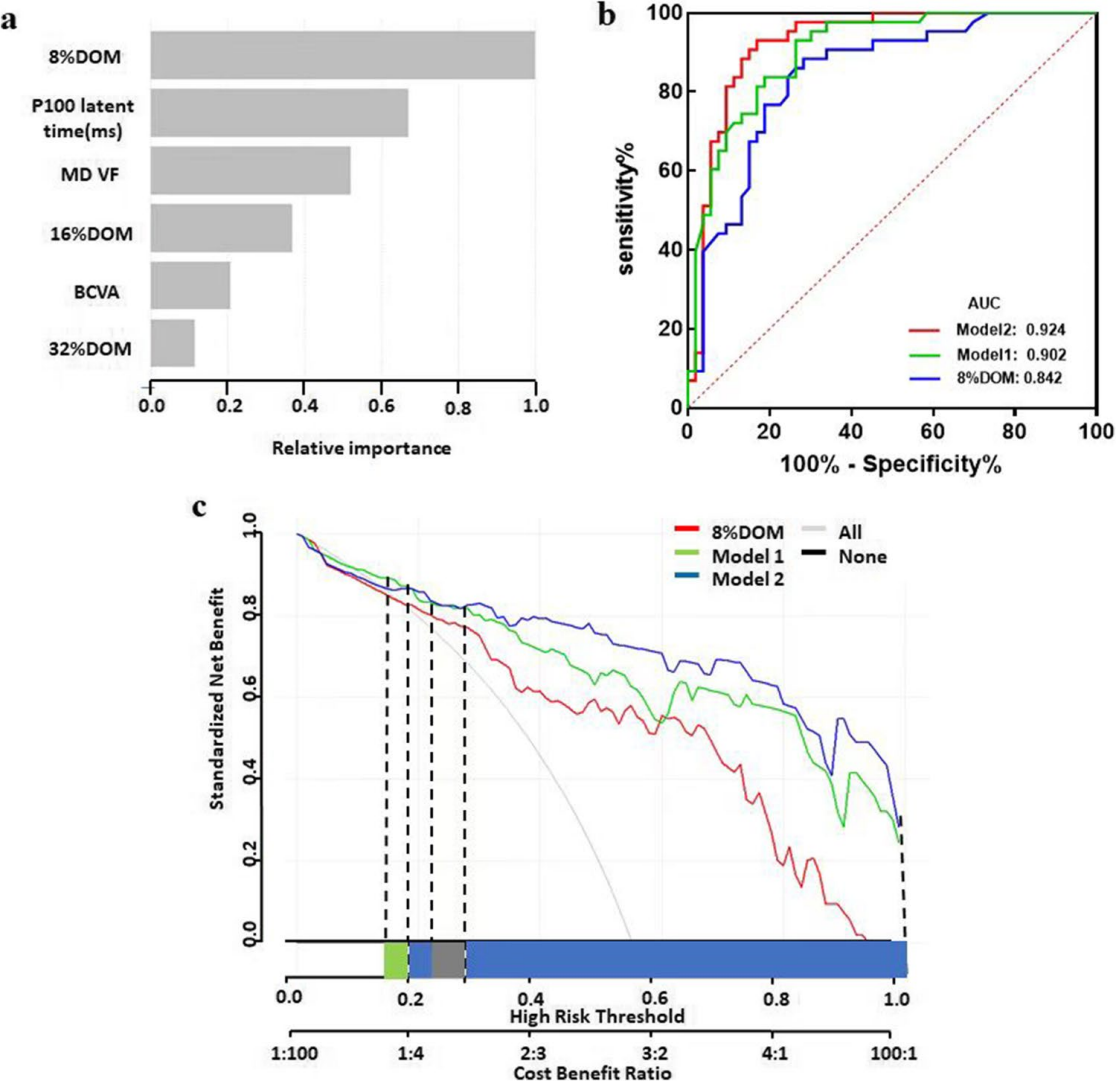
Comparison of the diagnostic capabilities of different diagnostic models for DON. Model 1 = 6.92785 − 2.45686*SNR(8%DOM) + 18.12121*( Papilledema = 1) − 5.03063*BCVA, Model 2 =  − 2.17895 − 1.73444* SNR(8%DOM) − 0.76181* SNR(16%DOM) + 0.06535* P100 latent time(ms) − 0.41904* MD(VF) − 3.61406* BCVA + 18.27022*(RAPD = 1), Machine and learning shows that the factors that affect DON were: SNR(8%DOM), P100 latent time (ms), MD (VF), SNR (16%DOM) and BCVA, Receiver operating characteristic curves for the models prediction of DON. The AUC values of model 2 (red curve) had the largest AUC (0.924) for discriminating DON from non-DONs. Decision curve analysis of three models for diagnosing DON. On the abscissa line, the blue square represents the high risk threshold range with highest NB of the blue line (model 2), and the green square represents the high risk threshold range of the green line (model 1) with highest NB, while the gray square indicates that multiple DCA curves partially overlap or basically overlap the high risk threshold range. The blue curve (model 2) has widest range with highest NB

In purpose of finding a more comprehensive, rapid and convenient method for predicting DON (Fig. 3a), we established two models to compare the diagnostic efficiency with that of icVEP (SNR of 8% DOM). Model 1 comprises an SNR value of 8% DOM of icVEP, papilledema, and BCVA. Model 2 includes SNR values of 8%, 16% DOM of icVEP, P100 latent time, MD (VF), BCVA, and RAPD. A ROC curve analysis (Fig. 3b) was used to evaluate the diagnostic efficiency of the above three methods. The AUC analysis (Supplementary Table 2) showed that Model 2 had the largest AUC (AUC = 0.924, cut-off =  − 0.085; Specificity = 0.930; Senstivity = 0.830) for discriminating DON from non-DONs, Model 1 had the second-largest AUC (AUC = 0.902; cut-off = 0.545; Specificity = 0.930; Senstivity = 0.736), and the SNR of 8% DOM had the smallest AUC (AUC = 0.842, cut-off = 0.910; Specificity = 0.884; Senstivity = 0.717). Although the AUC of Model 2 is larger than that of Model 1, they are not significantly different (*P* = 0.3992 > 0.05) (Supplementary Table 3). Furthermore, the AUCs of Model 1 and Model 2 are both significantly larger than that of 8% DOM (*p* = 0.0364 < 0.05; *p* = 0.0325 < 0.05) indicating that both Model 1 and Model 2 have no significantly different for diagnosing DON, and both of the above two models have better diagnostic efficiency compared with simply using the 8% DOM of icVEP.

We also applied DCA to the above three methods to diagnose DON from a clinical benefit perspective. DCA can identify risk models that can help us make better clinical decisions, when comparing two models, we check which model has the highest NB [1]. The NB curve is shown in Fig. 3c. The blue curve has the largest NB value within the maximum threshold range (blue square area), which means that the patients clinically benefit most from Model 2 when predicting DON compared to the other two. It has to be noted that, to the best of our knowledge, quantitative criteria for clinical diagnostic efficacy of DCA currently do not exist, and typically a qualitative determination of the diagnostic efficacy is provided from the DCA curve.

## Discussion

The present study demonstrated that icVEP has a promising capacity for discriminating between DON and non-DON eyes; moreover, it has greater sensitivity than pVEP and VF to detect the deficiencies of M-pathway in TAO eyes.

### icVEP is a potential technique for detecting DON

First, the icVEP was designed prone to record cortical activities by afferents in the M pathway, and studies based on glaucoma [11, 12, 14, 21] demonstrated that it can detect minor abnormalities sooner than any other technique since M cells have larger diameter and fewer numbers than the other cells in visual pathway, which make them vulnerable to damage [11, 22], leading us to speculate that icVEP might be a potential technique for detecting early stage of DON.

Second, there are several other functional techniques that provide insight into the state of the visual system, including pVEP, multifocal visual evoked potentials, and frequency-doubling technology; however, these either are time-consuming or subject to high individual variability [12, 21]. A single icVEP test can be completed in less than 2 min, which avoids the results being affected by excessive patient fatigue. Thus, the objective and efficient icVEP is expected to aid in detection of DON.

Third, icVEP can examine the functional integrity of central vision at all levels of the visual pathway[14]. In contrast, pattern electroretinogram can test only functional integrity at the retinal level [23], and optical coherence tomography can detect only morphological abnormalities at the retinal level without functional evidence. Thus, icVEP might be a particularly sensitive indicator of a functional deficit in the visual pathway prior to any observable structural damage [12].

### icVEP can distinguish DON eyes from non-DON eyes

We found that the SNRs obtained with icVEP were significantly reduced in patients with DON compared to non-DON, which is consistent with the previous diagnostic criteria of DON [7], it indicates that icVEP could be used as an alternative technique for diagnosing DON. It may due to the damage of the optic nerve caused by mechanical compression, stretching, and ischemia in DON, thus reducing the retinal transmission to the visual cortex, leading to a decrease in the SNR values of icVEP. The same phenomenon could also be observed in early open-angle glaucoma [24].

We also studied the factors associated with SNR and found that most of the visual function parameters were significantly correlated with the SNR values. More importantly, the negative correlation between SNR value and DON indicates that the results of icVEP could reflect the abnormality of visual function. These results further confirm that icVEP could be particularly helpful as an alternative technique for discriminating DON from non-DON.

### icVEP has better diagnostic potential when detecting DON than VF and pVEP

Our goal was to find a method with high sensitivity and specificity for diagnosing DON; therefore, the quantitative comparison between the diagnostic performance of icVEP and that of VF and pVEP was conducted using ROC curve analyses. The current findings suggest that the diagnostic power of icVEP is similar to that of the other two techniques for DON detection when the comparison was based solely on quantitative data.

In order to testify the clinical utility of these techniques, we applied DCA for further analysis. Although a model/technique with better discrimination and calibration should theoretically function as a better guide to clinical management, statistical measures (such as ROC curve analyses) fall short when we want to evaluate whether the model achieves clinical utility and improves clinical decision making [19]. To overcome this limitation, DCA was developed to summarize and integrate the preferences of patients or decision makers into the analysis, thus supporting decision-making. Our study shows that the parameters are quite well calibrated, achieving a relatively high AUC. More importantly, patients benefit most from using 8% DOM of icVEP during diagnosis.

In addition, we also found that though BCVA, VF, and pVEP of non-DON eyes did not change significantly when compared with controls, SNRs under 8% and 16% DOM showed significant differences, indicating that there might be subtle damages in the M-pathway during TAO (non-DON) stage, and icVEP could detect it earlier and more sensitively. Previous study [25] demonstrated that the contrast sensitivity at all spatial frequency of TAO patients without obvious DON was significantly worse than normal subjects, and a recent study [26] testified that mild TAO patients can have electrophysiological changes that might suggest neural changes in the early disease phase, our study further confirms their theories. Furthermore, we speculate that patients with TAO complained about blurred vision in the absence of decreased BCVA and dry eye, might be caused by early deficiency of M-pathway.

### The role of the multifactor combined diagnosis model in DON diagnosis

As mentioned above, statistical measures sometimes cannot be used exclusively to determine whether use of a model/technique is beneficial in making clinical decisions—especially when one model possesses better discrimination while the other possesses better calibration [27]. Therefore, based on our clinical diagnosis experience and previous study [18], we established two models, compared with using 8% DOM of icVEP, and calculated the ROC curves to confirm their diagnostic efficacy. The results suggest that both models are more effective in diagnosing DON than simply using 8% DOM of icVEP. Since Model 1 and Model 2 have no significant difference when diagnosing DON, we further applied DCA to identify which model might provide more clinical benefit for patients. The data suggest that patients benefit most from diagnosis using Model 2, which includes the most parameters.

It is understandable that the combination of more functional parameters that complement each other may greatly improve the ability to detect DON. However, completing all the tests in Model 2 is not only time-consuming but also makes it difficult for patients to cooperate and results in an increased financial burden. In contrast, the test in Model 1 is relatively simple, and time-efficient, and it offers high diagnostic accuracy. In general, when diagnosing DON, both Model 1 and Model 2 present effective diagnostic power depending on different clinical situations; thus, healthcare workers can comprehensively consider which diagnosis model would be most effective based on the disease development and other conditions, such as patients’ health status and economic situation.

## Limitations

With regard to limitations, this study still needs to be validated from more aspects. [1] Imaging examinations such as MRI can be used to further evaluate the anatomical damage of M-pathway during TAO development. [2] Though the whole detection process of icVEP takes only 2 s, and the detection is more objective, it only detects the function of the central retina but not the entire retina, other electrophysiological methods such as mfERG could be applied for future study. [3] In this study, we performed transient VEP(icVEP) to acquire electrical signals from the visual cortex, but it is unable to exclude influence of electrical activities from mental state, emotion, eye movement, and blinking from the subjects; thus, steady-state VEP could be applied in our future study to enhance the study. [4] The DON eyes in our study were not classified by severity, which would partly limit the clinical usefulness of this study. Though the BCVA as well as VF might reflect the severity of DON patients to some extent, the sample size of our study is limited. Thus, further expansion of the sample size and classified by severity in DON patients would greatly enhance the study. [5] Follow-up examinations are needed for comparing and verifying the diagnostic efficacy of icVEP.

## Conclusion

Our study suggests that the M-pathway might be damaged during DON development, even as early as TAO development, and the SNR of 8% DOM from icVEP is a potentially useful electrophysiological method to DON detection. Furthermore, icVEP combined with other clinical diagnostic tests should be considered as a diagnostic model in future clinical practice.

## Supplementary Information

Below is the link to the electronic supplementary material.

Supplementary file1 (PDF 522 KB)

## Abbreviations

DON: Dysthyroid optic neuropathy
TAO: Thyroid associated ophthalmopathy
CAS: Clinical activity score
dB: Decibel
PSD: Pattern standard deviation
VA: Visual acuity
BCVA: Best corrected visual acuity
RAPD: Relative afferent pupillary defect
IOP: Intraocular pressure
VF: Visual field
VEP: Visual evoked potential
pVEP: Pattern visual evoked potential
icVEP: Isolated – check visual evoked potential
M- pathway: Magnocellular pathway
DOM: Depth of modulation
SNR: Signal-to-noise ratio
AUC: Area under curve
DCA: Decision curve analysis
NB: Net benefit
SD: Standard deviation
I131 therapy: Radioiodine therapy
