# Correction to: Utility of isolated‑check visual evoked potential technique in dysthyroid optic neuropathy

**DOI:** 10.1007/s00417-023-05980-y

**Published:** 2023-01-27

**Authors:** Ban Luo, Rong Liu, Shanluxi Wang, Weikun Hu, Yunping Li, Boding Tong, Hong Zhang, Xin Qi

**Affiliations:** 1 Department of Ophthalmology, Tongji Hospital, Tongji Medical College, Huazhong University of Science and Technology, Qiaokou District, No.1095, Jiefang Avenue, Wuhan, 430030 Hubei China; 2 Department of Ophthalmology, Wenchang People’s Hospital, Wenchang, 571321 China; 3 Department of Ophthalmology, the Second Xiangya Hospital, Central South University, No.139, Renmin Middle Road, Changsha, 410011 Hunan China

**Correction to: Graefe's Archive for Clinical and Experimental Ophthalmology**

10.1007/s00417-023-05975-9

The article “Utility of isolated‑check visual evoked potential technique in dysthyroid optic neuropathy”, written by Ban Luo1, Rong Liu, Shanluxi Wang, Weikun Hu, Yunping Li, Boding Tong, Hong Zhang and Xin Qi, was originally published electronically on the publisher’s internet portal on 16 January 2023 without open access. With the author(s)’ decision to opt for Open Choice the copyright of the article changed on 23 January 2023 to © The Author(s) 2023 and the article is forthwith distributed under a Creative Commons Attribution 4.0 International License, which permits use, sharing, adaptation, distribution and reproduction in any medium or format, as long as you give appropriate credit to the original author(s) and the source, provide a link to the Creative Commons licence, and indicate if changes were made. The images or other third party material in this article are included in the article’s Creative Commons licence, unless indicated otherwise in a credit line to the material. If material is not included in the article’s Creative Commons licence and your intended use is not permitted by statutory regulation or exceeds the permitted use, you will need to obtain permission directly from the copyright holder. To view a copy of this licence, visit http://creativecommons.org/licenses/by/4.0.

The original article was corrected.

