# Supplementary material for: Utility of isolated-check visual evoked potential technique in dysthyroid optic neuropathy
Source: Graefes Arch Clin Exp Ophthalmol. 2023 Jan 16;261(7):2031–40. doi: 10.1007/s00417-023-05975-9 (PMC10271890; doi:10.1007/s00417-023-05975-9)
Supplement: Supplementary file 1 — Supplementary file1 (PDF 522 KB) [file 417_2023_5975_MOESM1_ESM.pdf]

Graefe's Archive for Clinical and Experimental Ophthalmology

Utility of isolated-check visual evoked potential technique in dysthyroid optic neuropathy

Ban Luo[1], Rong Liu[1]~~Hong Zhang[1]~~, Shanluxi Wang[1], Weikun Hu[1], Yunping Li[2], Boding Tong[2], Hong Zhang[1†]~~Rong Liu[1‡]~~, Xin Qi[2†]

1. Department of Ophthalmology, Tongji Hospital, Tongji Medical College, Huazhong University of Science and Technology, Wuhan, Hubei, China. 430030
2. Department of Ophthalmology, the Second Xiangya Hospital, Central South University, Changsha, Hunan, China. 410011

~~2.~~3. Department of Ophthalmology, Wenchang People's Hospital, Wenchang, China. 571321

Corresponding authos

Xin Qi: Department of Ophthalmology, The Second Xiangya Hospital, No.139, Renmin Middle Road, Changsha, 410011, Hunan Province, China.

E-mail: qixin78@csu.edu.cn

Telephone: +86-0731-85295137

Hong Zhang: Department of Ophthalmology, Tongji Hospital, No.1095, Jiefang Avenue, Qiaokou District, Wuhan, 430030, Hubei Province, China.

E-mail: zhanghong@vip.163.com~~zhanghong@vip.163.com~~

~~Rong Liu: Department of Ophthalmology, Tongji Hospital, No.1095, Jiefang Avenue, Qiaokou District, Wuhan, 430030, Hubei Province, China.~~

E-mail: 507531193@qq.com

Telephone: +86-027-83663411

Supplementary materials

eTable 1 Comparison of the DON diagnostic efficiency among the parameters

Variable	P value
8%DOM~PSD(VF)	0.7708
8%DOM~MD(VF)	0.6907
8%DOM~P100 latent time(ms)	0.3423
8%DOM~16%DOM	0.2567
8%DOM~32%DOM	0.1681
16%DOM~MD(VF)	0.6420
16%DOM~PSD(VF)	0.6163
16%DOM~P100 latent time(ms)	0.8815
16%DOM~32%DOM	0.8459
32%DOM~MD(VF)	0.5208
32%DOM~PSD(VF)	0.4770
32%DOM~P100 latent time(ms)	0.8815
P100 latent time(ms)~MD(VF)	0.4864
P100 latent time(ms)~PSD(VF)	0.5015
MD(VF)~PSD(VF)	0.9355

DOM, depth of modulation of the check luminance; PSD, pattern standard deviation; MD, mean deviation.

eTable 2 Diagnostic power of the models

	AUC	95%CI	Cut-off	Specificity	Sensitivity
Model1	0.902	0.843-0.962	0.545	0.930	0.736
Model 2	0.924	0.868-0.980	-0.085	0.930	0.830
SNR(8%DOM)	0.842	0.763-0.922	0.910	0.884	0.717

Model 2 has the largest AUC for discriminating DON from non-DONs, Model 1 had the second large AUC; SNR of 8%DOM had the smallest AUC.

eTable 3 Comparison of the DON diagnostic efficiency among the models

Variable	P value
8%DOM~Model 1	**0.0364**
8%DOM~Model 2	**0.0325**
Model 1~Model 2	0.3992

Values with statistical significance (P<0.05) are in bold.
